# New apotirucallane-type triterpenoids from *Chisocheton paniculatus*

**DOI:** 10.1007/s13659-012-0065-5

**Published:** 2012-12-12

**Authors:** Feng Zhang, Xiu-Feng He, Wen-Bin Wu, Wan-Sheng Chen, Jian-Min Yue

**Affiliations:** 1 Department of Pharmacy, Changzheng Hospital, Second Military Medical University, 415 Fengyang Road, Shanghai, 200003 China; 2 State Key Laboratory of Drug Research, Institute of Materia Medica, Shanghai Institutes for Biological Sciences, Chinese Academy of Sciences, 555 Zuchongzhi Road, Zhangjiang Hi-Tech Park, Shanghai, 201203 China

**Keywords:** *Chisocheton paniculatus*, apotirucallane triterpenoids, chisiamols G and H

## Abstract

Two new apotirucallane triterpenoids, namely chisiamols G (**1**) and H (**2**), featuring a 21,23-lactone, together with five known triterpenoids, were isolated from the twigs of *Chisocheton paniculatus*. The structures of the new compounds were elucidated on the basis of spectroscopic and chemical methods.

**Electronic Supplementary Material:**

Supplementary material is available for this article at 10.1007/s13659-012-0065-5 and is accessible for authorized users.

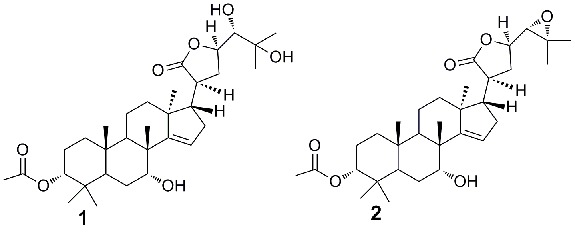

## Introduction

The genus *Chisocheton* (Meliaceae) comprising about 50 species is mainly distributed in India and Malaysia, which is traditionally used to treat stomach and kidney problems, backache, fever, rheumatism and malaria. *Chisocheton paniculatus* is the only species growing in southern China.[1] Phytochemical studies of genus *Chisocheton* have led to the isolation of sesquiterpenoids, triterpenoids and limonoids, some of which showed anti-inflammatory, anti-feedant, anti-fungal and cytotoxic activities. Previous phytochemical investigation on *C. paniculatus* resulted mainly in the isolation of apotirucallane-type triterpenoids.[2] In the present study, two new apotirucallane triterpenoids, named chisiamols G (**1**) and H (**2**), together with five known triterpenoids, chisiamol A (**3**),[2m] sapelin B (**4**),[3] 3$\alpha$-acetoxy-21,24*R*-epoxyapotirucall-14-ene-7$\alpha$,23*R*,25-triol (**5**),[2m] chisiamol B (**6**),[2m] and 3$\alpha$-acetoxy-21,23-epoxyapotirucall-14-ene-7$\alpha$,21*R*,24,25-tetrol (**7**)[2m] were isolated from the leaves and stems of *C. paniculatus* (Figure 1). Herein, we describe the isolation and structural elucidation of these new compounds.

**Figure 1.** Chemical structures of compounds **1**–**7**

## Results and Discussion

Compound **1** was obtained as a white amorphous powder. Its molecular formula was determined to be $C_{32}H_{50}O_7$ by HRESIMS *m*/*z* $[M + Na]^+$ 569.3458 (calcd for $C_{32}H_{50}O_7Na$, 569.3454). The IR absorptions at 3448, 1720 and 1630 $cm^{-1}$ indicated the presence of hydroxyl, ester carbonyl, and double bond groups, respectively. The $^1H$ NMR spectrum showed seven tertiary methyls at $\delta_H$ 0.85, 0.93, 0.96, 1.09, 1.12, 1.24, and 1.28 (each 3H, s), one acetyl methyl at $\delta_H$ 2.06 (3H, s), one olefinic proton at $\delta_H$ 5.45, and four proton signals at $\delta_H$ 3.26 (d), 3.95 (br. s), 4.66–4.74 (m), and 4.63 (br. s) attributable to the protons of oxygenated methines (Table 1). All the 32 carbon resonances were resolved in the $^{13}C$ NMR spectrum, and further classified by DEPT and HSQC experiments as eight methyls, seven $sp^3$ methylenes, eight $sp^3$ methines (four oxygenated), five $sp^3$ quaternary carbons (one

*To whom correspondence should be addressed. E-mail: chenwanshengsmmu@yahoo.com.cn (W.S. Chen); jmyue@mail.shcnc.ac.cn (J.M. Yue)

**Table 1.** $^{1}$**H (400 MHz) and** $^{13}$**C (100 MHz) NMR data of 1 and 2 (CD$_3$OD)**

| position | chisiamol G (**1**) | | chisiamol H (**2**) | |
|---|---|---|---|---|
| | $\delta_H$ | $\delta_C$ | $\delta_H$ | $\delta_C$ |
| 1$\alpha$ | 1.22–1.30, m | 35.0, CH$_2$ | 1.25–1.32, m | 35.0, CH$_2$ |
| 1$\beta$ | 1.33–1.42, m | | 1.36–1.46, m | |
| 2$\alpha$ | 1.50–1.57, m | 24.3, CH$_2$ | 1.52–1.62, m | 24.3, CH$_2$ |
| 2$\beta$ | 1.87–1.99, m | | 1.91–1.98, m | |
| 3 | 4.63, br. s | 80.3, CH | 4.62, br. s | 80.3, CH |
| 4 | | 37.8, C | | 37.8, C |
| 5 | 1.97–2.09, m | 43.6, CH | 1.99–2.11, m | 43.6, CH |
| 6$\alpha$ | 1.61–1.67, m | 26.0, CH$_2$ | 1.62–1.73, m | 26.0, CH$_2$ |
| 6$\beta$ | 1.72–1.80, m | | 1.76–1.83, m | |
| 7 | 3.95, br. s | 74.4, CH | 3.95, br. s | 74.4, CH |
| 8 | | 45.7, C | | 45.7, C |
| 9 | 1.97–2.09, m | 43.7, CH | 1.99–2.11, m | 43.7, CH |
| 10 | | 39.3, C | | 39.3, C |
| 11$\alpha$ | 1.48–1.53, m | 18.0, CH$_2$ | 1.53–1.58, m | 18.0, CH$_2$ |
| 11$\beta$ | 1.67–1.73, m | | 1.71–1.77, m | |
| 12 | 2.09–2.18, m | 34.3, CH$_2$ | 2.15–2.22, m | 34.4, CH$_2$ |
| 13 | | 48.5, C | | 48.5, C |
| 14 | | 162.2, C | | 162.3, C |
| 15 | 5.45, d (1.7) | 120.8, CH | 5.46, d (2.3) | 120.6, CH |
| 16$\alpha$ | 2.28–2.35, m | 33.6, CH$_2$ | 2.28–2.34, m | 33.8, CH$_2$ |
| 16$\beta$ | 1.99–2.08, m | | 2.04–2.12, m | |
| 17 | 2.15–2.22, m | 56.2, CH | 2.15–2.22, m | 56.1, CH |
| 18 | 1.09, s | 20.8, CH$_3$ | 1.09, s | 20.7, CH$_3$ |
| 19 | 0.96, s | 16.4, CH$_3$ | 0.96, s | 16.4, CH$_3$ |
| 20 | 2.81–2.90, m | 41.6, CH | 2.95, ddd (5.7, 8.4, 12.0) | 41.6, CH |
| 21 | | 181.6, C | | 181.0, C |
| 22$\alpha$ | 2.18–2.33, m | 32.5, CH$_2$ | 2.45, ddd (6.5, 8.6, 12.6) | 31.7, CH$_2$ |
| 22$\beta$ | 2.18–2.33, m | | 1.99–2.06, m | |
| 23 | 4.66–4.74, m | 79.1, CH | 4.26, ddd (5.7, 7.4, 10.5) | 80.6, CH |
| 24 | 3.26, d (1.9) | 79.5, CH | 2.86, d (8.1) | 66.4, CH |
| 25 | | 73.9, C | | 59.4, C |
| 26 | 1.24, s | 25.3, CH$_3$ | 1.34, s | 19.9, CH$_3$ |
| 27 | 1.28, s | 28.5, CH$_3$ | 1.34, s | 25.4, CH$_3$ |
| 28 | 0.93, s | 22.8, CH$_3$ | 0.92, s | 22.8, CH$_3$ |
| 29 | 0.85, s | 28.6, CH$_3$ | 0.85, s | 28.6, CH$_3$ |
| 30 | 1.12, s | 29.3, CH$_3$ | 1.10, s | 29.3, CH$_3$ |
| 3-OAc | | 173.2, C | | 173.2, C |
| | 2.06, s | 21.7, CH$_3$ | 2.06, s | 21.7, CH$_3$ |

oxygenated), two ester carbonyls ($\delta_C$ 173.2 and 181.6), and one trisubstituted double bond ($\delta_C$ 120.8 and 162.2) (Table 1). Careful analysis of its $^{1}$H and $^{13}$C NMR data indicated that the NMR data of **1** highly resembled those of the co-existing compound 3$\alpha$-acetoxy-21,23-epoxyapotirucall-14-ene-7$\alpha$,21*R*,24,25-tetrol (**7**)[2m] except for the absence of both the proton and carbon signals of the hemiacetal group ($\delta_C$ 96.5; $\delta_H$ 5.29), and the presence of an additional ester carbonyl, suggesting that C-21 hemiacetal in **7** was oxygenated to a lactone group ($\delta_C$ 181.6) in **1**. To prove this speculation, an HMBC experiment was performed, in which, the ester carbonyl was placed at C-21 by the correlations from H$_2$-22 ($\delta_H$ 2.18–2.33) and H-20 ($\delta_H$ 2.81–2.90) to C-21 ($\delta_C$ 181.6). This was supported by $^{1}$H-$^{1}$H COSY spectrum, in which, a proton bearing structural fragment from C-17 to C-24 as depicted in bold bond (Figure 2) was revealed. In addition, the key HMBC correlations from H-3 ($\delta_H$ 4.63) to C-1′ ($\delta_C$ 173.2) assigned an acetoxy group at C-3; the HMBC correlations of Me-30 ($\delta_H$ 1.12)/C-7 ($\delta_C$ 74.4), and H-7 ($\delta_H$ 3.95)/C-6 located a hydroxyl group at C-7 (Figure 2).

In the ROESY spectrum of **1**, the correlations of Me-19/H-3, Me-29/H-3, Me-19/Me-30, and Me-30/H-7, indicated that Me-19, Me-29, Me-30, H-3, and H-7 were cofacial and were randomly assigned to be $\beta$-oriented. Subsequently, the ROESY correlations of H-20/H-23, and Me-18/H-20 indicated that they were $\alpha$-oriented (Figure 4). The small coupling constant (1.9 Hz) between H-23 and H-24 indicated that they were in a *gauche* relationship, showing that H-24 was $\beta$-oriented.[4]

**Figure 2.** $^{1}$H-$^{1}$H COSY (bold lines) and key HMBC (H→C) correlations of **1** and **2**

The structure of compound **1** was further verified by chemical correlation with the coexisting known compound **7**. Both compounds **1** and **7** were treated with LiAlH$_4$ to afford a common compound **8** (Figure 3),[5] confirming the structural assignment for compound **1**. The structure of **8** was assigned as the reduced derivative of compound **7** based on the spectroscopic data. In the ESIMS, the [M + Na]$^+$ peak at *m/z* 531.4, [2M + Na]$^+$ peak at *m/z* 1039.7 and [M + HCOO]$^-$ peak at *m/z* 553.7 were all consistent with the molecular formula $C_{30}H_{52}O_6$ of **8** with six hydroxyl groups. A comparison of $^1$H NMR spectra of **7** and **8** was particularly informative. The absence of the acetyl methyl ($\delta_H$ 2.06 in **7**) and the upfield shifted H-3 at $\delta_H$ 3.33 ($\delta_H$ 4.63 in **7**) of **8** indicated the presence of OH-3 in **8**. The remaining two hydroxyls in **8** were assigned to C-21 and C-23 by the chemical shifts of $H_2$-21 at $\delta_H$ 3.45 and $\delta_H$ 3.77 (each 1H, $J$ = 11.0 Hz) and H-23 at $\delta_H$ 4.08, respectively.

**Figure 3.** Reduction of compounds **1** and **7** to afford **8**

Hence, the structure of **1**, namely chisiamol G, was assigned as 3$\alpha$-acetoxy-7$\alpha$,24,25-trihydroxyl-21,23-epoxyapotirucall-14-ene-21-one, an apotirucallane-type triterpenoid possessing a five-membered lactone formed between C-21 and C-23.

Compound **2** was obtained as a white amorphous powder. Its molecular formula $C_{32}H_{48}O_6$ was assigned by HRESIMS at *m*/*z* $[M + Na]^+$ 551.3348 (calcd for $C_{32}H_{48}O_6Na$, 551.3349). Its IR spectrum exhibited absorption bands for hydroxyl (3432 $cm^{-1}$), ester carbonyl (1724 $cm^{-1}$), and double bond (1630 $cm^{-1}$) functionalities. A comparison of the $^{13}$C NMR spectra (Table 1) of compounds **1** and **2** revealed that their structures were close related, except for that two oxygenated carbon signals at $\delta_C$ 79.5 (C-24) and at $\delta_C$ 73.9 (C-25) in **1** were significantly upfield shifted to $\delta_C$ 66.4 (C-24) and $\delta_C$ 59.4 (C-25) in **2**, respectively**,** suggesting that an 24,25-epoxide ring was present in **2** instead of the 24,25-diol motif in **1**. This was consistent with the fact that the molecular weight of **2** was 18 mass units less than that of **1**, and was further supported by the almost identical chemical shifts of C-24 and C-25 of **2** as compared with those of 7$\alpha$-hydroxyl-21$\alpha$-acetoxy-21,23-epoxyapotirucall-24,25-epoxide-14-ene-3-one and 3$\alpha$,7$\alpha$-di-hydroxyl-21$\alpha$-acetoxy-21,23-epoxyapotirucall-24,25-epoxide-14-ene, both of which possessing an 24,25-epoxide were respectively reported as compounds A and D in the literature.[2a] The structure of **2** was finally confirmed by 2D NMR spectra, especially HMBC (Figure 2) and ROESY (Figure 4) spectra. In the ROESY experiment, the correlations of Me-29/H-3, Me-19/Me-30, and Me-30/H-7, indicated that Me-19, Me-29, Me-30, H-3, and H-7 were cofacial and were randomly assigned to be $\beta$-oriented. Furthermore, the ROESY correlations of H-20/H-23, and Me-18/H-20 indicated that Me-18, H-20, and H-23 were cofacial and $\alpha$-oriented. The 24,25-epoxide ring was assigned to be $\alpha$-directed by the ROESY correlations of H-22$\alpha$/H-23 and H-22$\beta$/H-24 (Figure 4).[6] The chemical shifts of C-24 ($\delta_C$ 66.4) and C-25 ($\delta_C$ 59.4), and the coupling constant ($J_{23,24}$ = 8.1 Hz) between H-23$\alpha$ and H-24 further supported the $\alpha$-orientation of the epoxide in **2** as compared with the corresponding data of the bruceajavanones A–E.[6] The structure of **2** was thus established as 3$\alpha$-acetoxy-7$\alpha$-hydroxyl-21,23-epoxyapotirucall-24,25-epoxide-14-ene-21-one, namely chisiamol H.

**Figure 4.** Key NOE (H↔H) correlations of **1** and **2**

All isolated compounds (**1**–**7**) were tested in an anti-microbial assay against *Helicobacter pylori*-SS1 *in vitro* according to standard protocols, in which Minimum inhibitory concentration (MIC) > 100 $\mu$g/mL was defined as inactive[7], and metronidazole (Shanghai Henshan Pharmaceutical Company, Ltd. with a purity of 99%; MIC = 0.312 $\mu$g/mL) was used as the positive control. However, none of these compounds showed activity (MIC > 50 $\mu$g/mL).

## Experimental Section

**General Experimental Procedures.** Optical rotations were measured with a Perkin-Elmer 341 polarimeter. UV spectra were measured on a Shimadzu UV-2550 spectrophotometer. IR spectra were recorded on a Perkin-Elmer 577 spectrometer using KBr disks. NMR spectra were measured on a Bruker AM-400 spectrometer. ESIMS was carried out on a Finnigan LCQ-DECA instrument, and HRESIMS spectra were made on a Bruker Daltonics micrOTOFQII or a Waters-Micromass Q-TOF Ultima Global electrospray mass spectrometer. All solvents used were of analytical grade (Shanghai Chemical Reagents Company, Ltd.). Different absorbents were employed for column chromatography: silica gel (200–300 mesh) and silica gel H (Qingdao Haiyang Chemical Co. Ltd.); RP-18 reversed silica gel (150–200 mesh, Merck); MCI gel (CHP20P, 75–150 ím, Mitsubishi Chemical Industries Ltd.); Sephadex LH-20 gel (Amersham Biosciences). Fractions were monitored by TLC (silica gel GF254, 0.25 mm, Merck), and spots were visualized by heating silica gel plates sprayed with 10% $H_2SO_4$ in ethanol. Semipreparative HPLC was performed on a Waters 1525 pump equipped with a Waters 2489 detector and an YMC-Pack ODS-A column (10 × 250 mm, S-5 $\mu$m, 12 nm).

**Plant Materal.** The leaves and stems of *C. paniculatus*, were collected in Mengla County, Yunnan Province of China in September of 2009, and the plant was identified by Professor You-Kai Xu of Xishuangbanna Tropical Botanical Garden, Chinese Academy of Sciences. A voucher specimen (accession number CS-2009-2Y) has been deposited in the Shanghai Institute of Materia Medica, Chinese Academy of Sciences.

**Extraction and Isolation.** Dried leaves and stems of *C. paniculatus* (6 kg) were extracted by 95% EtOH at room temperature. The crude extract (70 g) was dissolved in $H_2O$ (500 mL) and then extracted with EtOAc (200 mL × 3). The EtOAc extract (24 g) was subjected to an MCI column (75–150 $\mu$m, 8 × 30 cm, MeOH/$H_2O$, 50:50 to 90:10, v/v) to give fractions E–K. Fraction I (1.80 g) was chromatographed on a silica gel column eluted with petroleum ether/$Me_2CO$ (300–400 mesh, 4 × 20 cm, 10:1 to 4:1) to yield four fractions (I1–4). Fraction I4 (193 mg) was subjected to a silica gel column (300–400 mesh, 3 × 18 cm, $CH_2Cl_2$/MeOH, 150:1, 100:1, 70:1, 50:1, 30:1, 20:1, 10:1) to give four fractions (I4a–d). Purification of fraction I4b (30 mg) by a RP-18 silica gel column (150–200 mesh, 3 × 15 cm, MeOH/$H_2O$, 70:30 to 80:20) gave **1** (20 mg). Fraction J (5.50 g) was chromatographed on a silica gel column eluted with petroleum ether/$Me_2CO$ (300–400 mesh, 8 × 20 cm, 10:1 to 6:1) to yield seven fractions (J1–7). Fraction J3 (221 mg) was submitted to a silica gel column (300–400 mesh, 3 × 20 cm, $CH_2Cl_2$/MeOH, 100:1, 80:1, 60:1) to give three fractions (J3a–c). Purification of fraction J3a (12 mg) with Sephadex LH-20 (3 × 100 cm, EtOH) yielded **2** (3 mg). Fraction J3b (73 mg) was applied to a RP-18 silica gel column (150–200 mesh, 3 × 15 cm, MeOH/$H_2O$, 80:20 to 90:10) to give fractions J3b1 and J3b2. Fraction J3b2 (14 mg) was further purified by preparative HPLC (eluting with MeCN/$H_2O$, 85:15 to 95:5, from 0 to 20 min, 3 mL/min) afforded **3** (3 mg). Purification of fraction J3c (15 mg) by preparative HPLC (eluting with MeCN/$H_2O$, 90:10 to 95:5, from 0 to 20 min, 3 mL/min) yielded **4** (4 mg). Fraction J5 (358 mg) and J6 (293 mg) were subjected to a silica gel column (300–400 mesh, 3 × 20 cm, petroleum ether/ EtOAc, 10:1, 8:1, 6:1, 4:1) to give two corresponding fractions J5a–b and J6a–b, respectively. Fraction J5a (140 mg) and J6a (255 mg) were applied to a silica gel column (300–400 mesh, 3 × 18 cm, $CH_2Cl_2$/MeOH, 100:1, 80:1, 60:1, 40:1) to give three corresponding fractions J5a1–3 and J6a1–3, respectively. Purification of fractions J5a2 (40 mg) and J6a3 (83 mg) were subjected to RP-18 silica gel column (150–200 mesh, 3 × 15 cm, MeOH/$H_2O$, 90:10 to 100:0) to afford **5** (35 mg) and **6** (9 mg). Fraction E (1.34 g) was chromatographed on a silica gel column eluted with petroleum ether/$Me_2CO$ (300–400 mesh, 6 × 20 cm, 10:1 to 1:1) to yield seven fractions (E1–7). Purification of fraction E3 (40 mg) was applied to RP-18 silica gel column (150–200 mesh, 3 × 15 cm, MeOH/$H_2O$, 80:20 to 90:10) to afford **7** (20 mg).

**Chisiamol G (1):** White amorphous powder; $[\alpha]_D^{23}$ – 105 (*c* = 0.27, MeOH); UV (MeOH) $\lambda_{max}$ (log ε) 202.4 (4.3) nm; IR (KBr) $\nu_{max}$ 3448, 2939, 2871, 1765, 1720, 1630, 1458, 1385, 1169, 1034 $cm^{-1}$; $^1H$ NMR and $^{13}C$ NMR see Table 1; ESIMS *m/z* $[M + Na]^+$ 569.4; HRESIMS *m/z* $[M + Na]^+$ 569.3458 (calcd for $C_{32}H_{50}O_7Na$, 569.3454).

**Chisiamol H (2):** White amorphous powder; $[\alpha]_D^{23}$ – 69 (*c* = 0.30, MeOH); UV (MeOH) $\lambda_{max}$ (log ε) 201.6 (4.15) nm; IR (KBr) $\nu_{max}$ 3432, 2937, 2871, 1774, 1724, 1630, 1458, 1385, 1250, 1188, 1024, 818 $cm^{-1}$; $^1H$ NMR and $^{13}C$ NMR see Table 1; ESIMS *m/z* $[M + Na]^+$ 551.4; HRESIMS *m/z* $[M + Na]^+$ 551.3348 (calcd for $C_{32}H_{48}O_6Na$, 551.3349).

**Reduction of Compounds 1 and 7 with $LiAlH_4$.** To a cooled solution (– 78 °C) of compound **1** or **7** (5.0 mg) in dry THF (2 mL), $LiAlH_4$ (3 mg) was added under nitrogen atmosphere. After stirring at the same temperature for 2 h, the reaction mixture was then allowed to r.t. and kept stirring overnight. After workup, the reaction mixture was diluted with $H_2O$ (2 mL) and then extracted with EtOAc (3 × 5 mL). The combined organic phase were dried with anhydrous $Na_2SO_4$ and concentrated *in vacuo* to obtain the crude product, which was purified by a silica gel column eluted with $CH_2Cl_2$/MeOH (300–400 mesh, 3 × 12 cm, 50:1 to 10:1) to give compound **8** (about 3.0 mg).

Compound **8**: ESIMS *m/z* 531.4 $[M + Na]^+$, *m/z* 1039.7 $[2M + Na]^+$, *m/z* 553.7 $[M + HCOO]^-$; $^1H$ NMR (400 MHz, $\delta_H$ ppm, $CD_3OD$): 5.44 (H-15, d, *J* = 2.6 Hz), 4.08 (H-23, t, *J* = 6.7), 3.88 (H-17, t-like), 3.45, 3.77 (H-21, ABq, *J* = 11.0 Hz), 3.33 (H-3, t-like), 3.12 (H-24, d, 1.5 Hz).

## Electronic Supplementary Material

Supplementary material is available in the online version of this article at http://dx.doi.org/ 10.1007/s13659-012-0065-5 and is accessible for authorized users.

## Acknowledgments

Financial support of the National Natural Science Foundation (Grant No. 21072203) and National Science and Technology Major Project "Key New Drug Creation and Manufacturing Program" (No. 2011ZX09307-002-03) of China is gratefully acknowledged. We thank Prof. You-Kai Xu for the identification of the plant material.

**Open Access** This article is distributed under the terms of the Creative Commons Attribution License which permits any use, distribution, and reproduction in any medium, provided the original author(s) and source are credited.

## Electronic supplementary material

Supplementary material, approximately 3.74 MB.
