# Supplementary material for: New apotirucallane-type triterpenoids from Chisocheton paniculatus
Source: Nat Prod Bioprospect. 2012 Dec 12;2(6):235–9. doi: 10.1007/s13659-012-0065-5 (PMC4131609; doi:10.1007/s13659-012-0065-5)
Supplement: Supplementary file 1 — Supplementary material, approximately 3.74 MB. [file 13659_2012_65_MOESM1_ESM.pdf]

Electronic Supplementary Material

New apotirucallane-type triterpenoids from *Chisocheton paniculatus*

Feng ZHANG,[a,b] Xiu-Feng HE,[b] Wen-Bin WU,[b] Wan-Sheng CHEN,[a,]* and Jian-Min YUE[b,]*

[a]Department of Pharmacy, Changzheng Hospital, Second Military Medical University, 415 Fengyang Road, Shanghai 200003, China

[b]State Key Laboratory of Drug Research, Institute of Materia Medica, Shanghai Institutes for Biological Sciences, Chinese Academy of Sciences, 555 Zuchongzhi Road, Zhangjiang Hi-Tech Park, Shanghai 201203, China

Received 5 August 2012; Accepted 7 October 2012

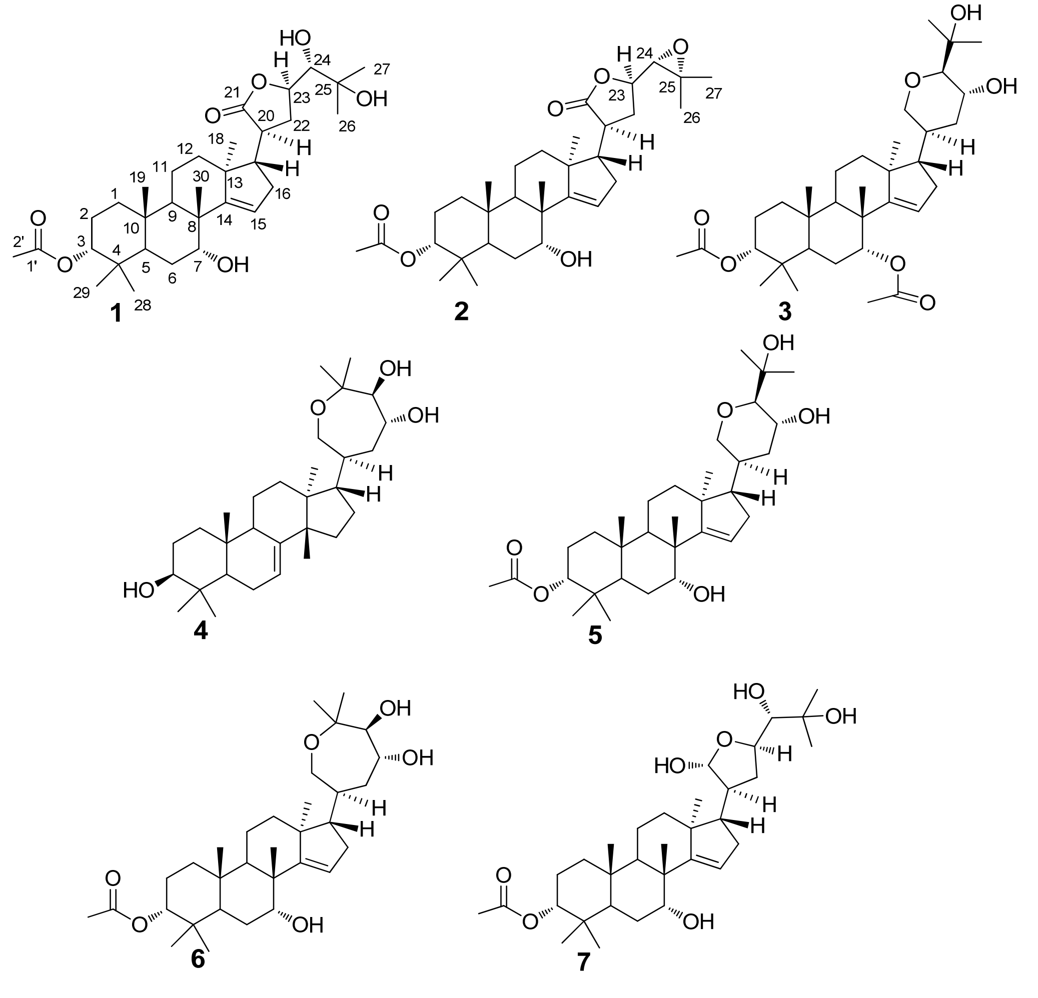

Structures of compounds **1**–**7**

*To whom correspondence should be addressed. E-mail: chenwanshengsmmu@yahoo.com.cn (W.S. Chen); jmyue@mail.shcnc.ac.cn (J.M. Yue)

For Compound **1**

S1. ^{1}H spectrum of chisiamol G (**1**) in CD_3OD
S2. ^{13}C spectrum of chisiamol G (**1**) in CD_3OD
S3. HSQC spectrum of chisiamol G (**1**) in CD_3OD
S4. HMBC spectrum of chisiamol G (**1**) in CD_3OD
S5. COSY spectrum of chisiamol G (**1**) in CD_3OD
S6. ROESY spectrum of chisiamol G (**1**) in CD_3OD
S7. ESIMS spectrum of chisiamol G (**1**)
S8. HRESIMS spectrum of chisiamol G (**1**)
S9. IR spectrum of T chisiamol G (**1**)

For Compound **2**

S10. ^{1}H spectrum of chisiamol H (**2**) in CD_3OD
S11. ^{13}C spectrum of chisiamol H (**2**) in CD_3OD
S12. HSQC spectrum of chisiamol H (**2**) in CD_3OD
S13. HMBC spectrum of chisiamol H (**2**) in CD_3OD
S14. COSY spectrum of chisiamol H (**2**) in CD_3OD
S15. ROESY spectrum of chisiamol H (**2**) in CD_3OD
S16. ESIMS spectrum of chisiamol H (**2**)
S17. HRESIMS spectrum of chisiamol H (**2**)
S18. IR spectrum of chisiamol H (**2**)

S19. ^{1}H spectrum of compound **8** in CD_3OD
S20. ESIMS spectra of compound **8**

S1. ^{1}H spectrum of chisiamol G (**1**) in CD_3OD

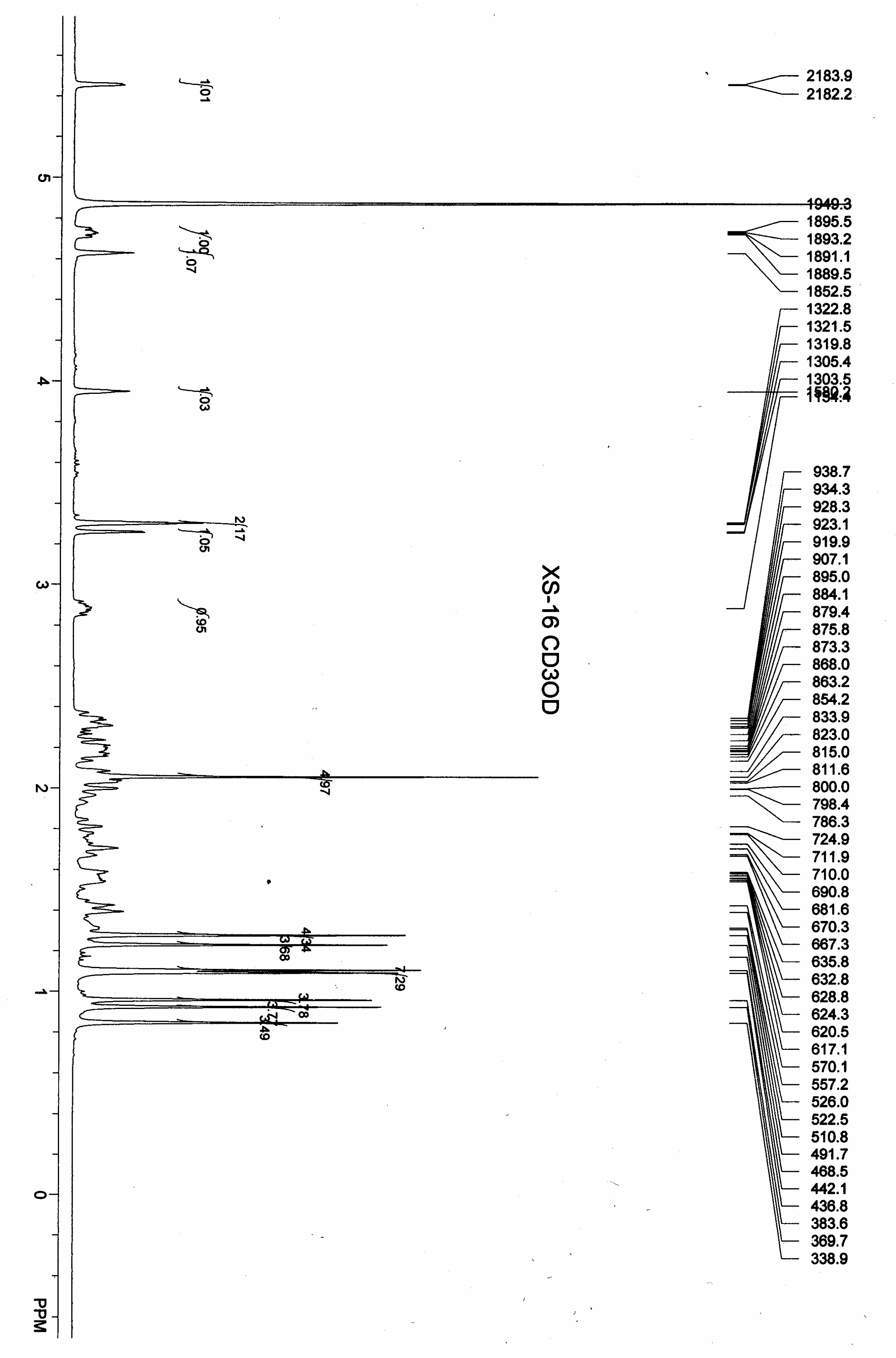

S2. ^{13}C spectrum of chisiamol G (**1**) in CD_3OD

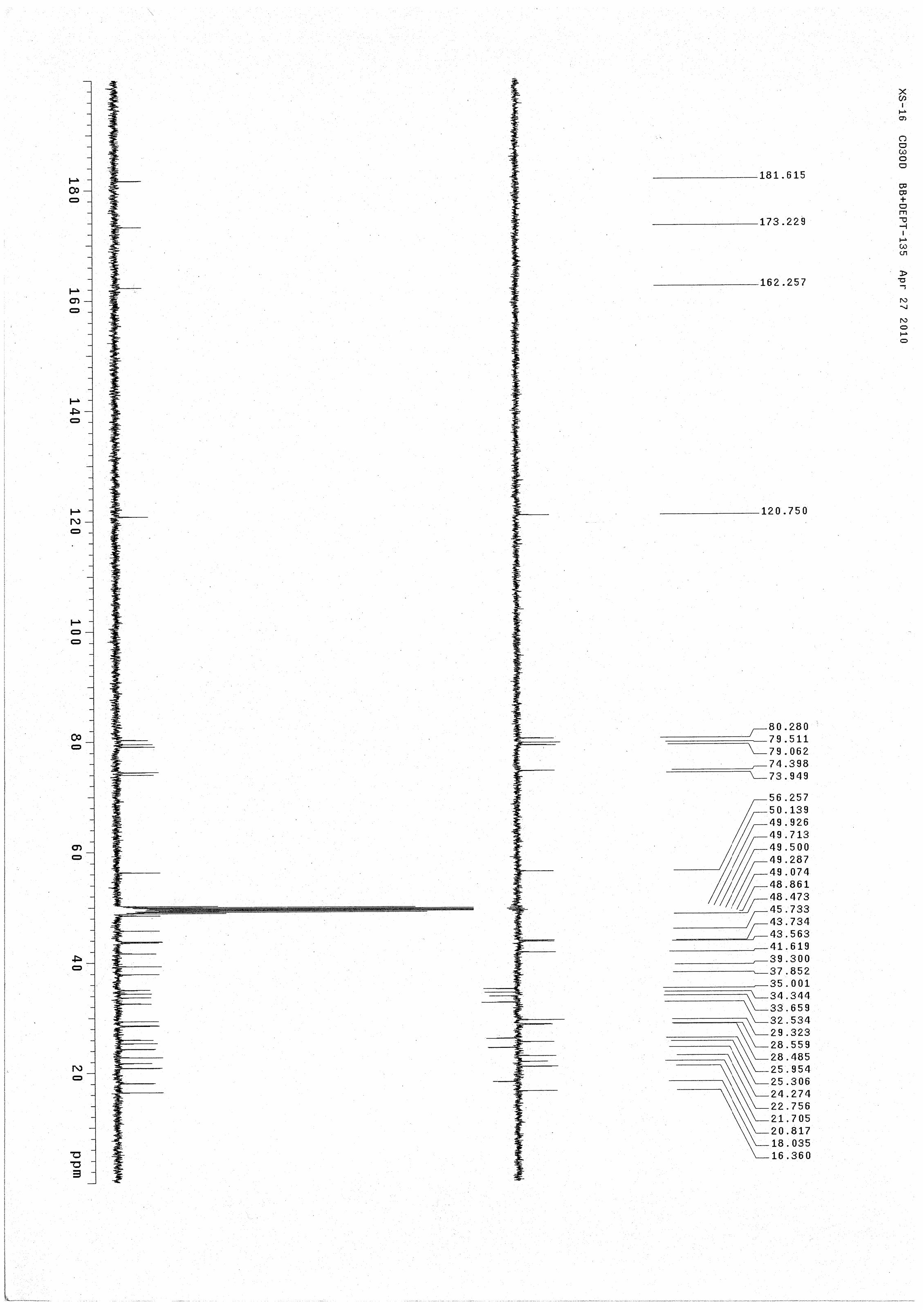

S3. HSQC spectrum of chisiamol G (**1**) in CD_3OD

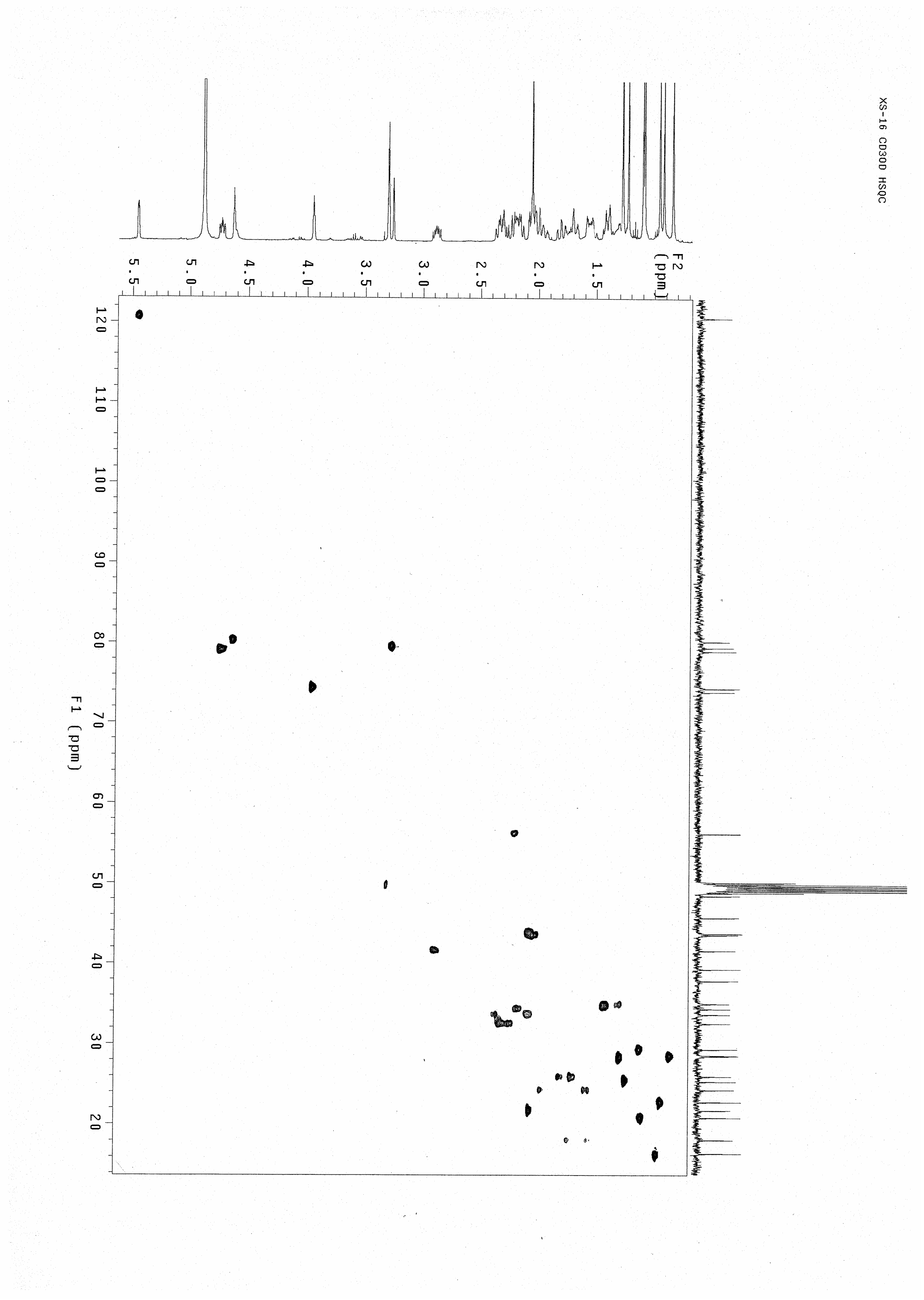

S4. HMBC spectrum of chisiamol G (**1**) in CD_3OD

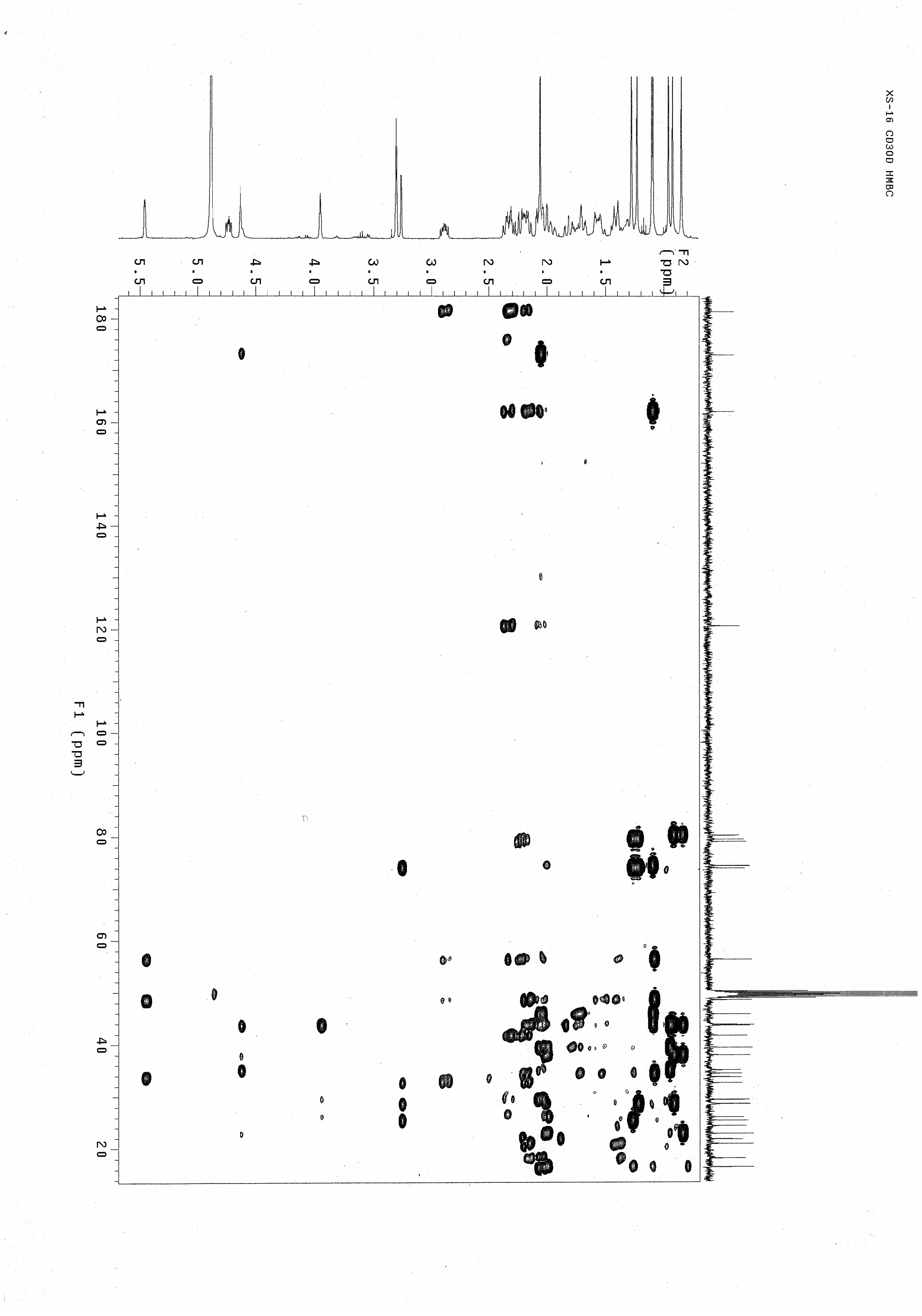

S5. COSY spectrum of chisiamol G (**1**) in CD_3OD

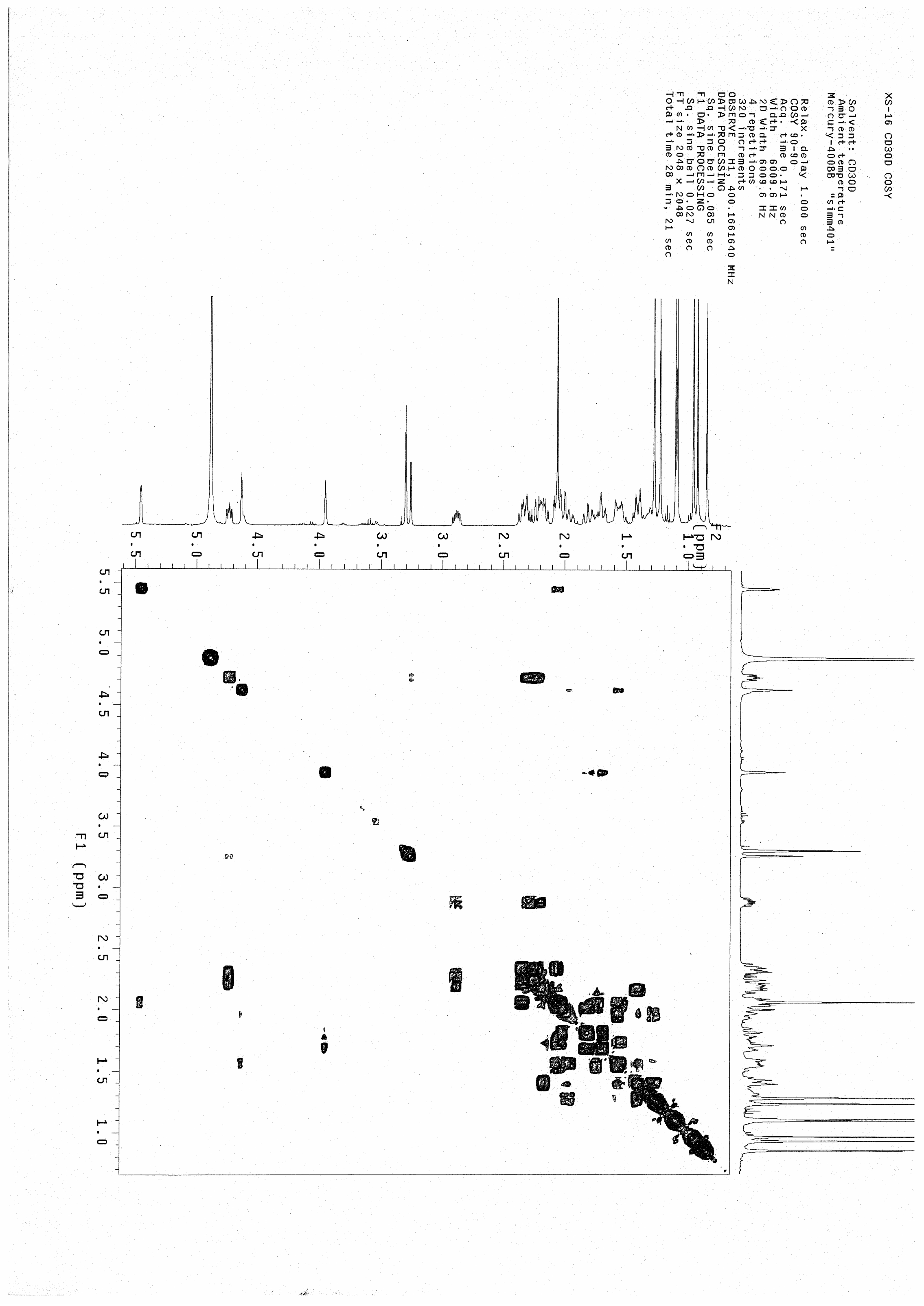

S6. ROESY spectrum of chisiamol G (**1**) in CD_3OD

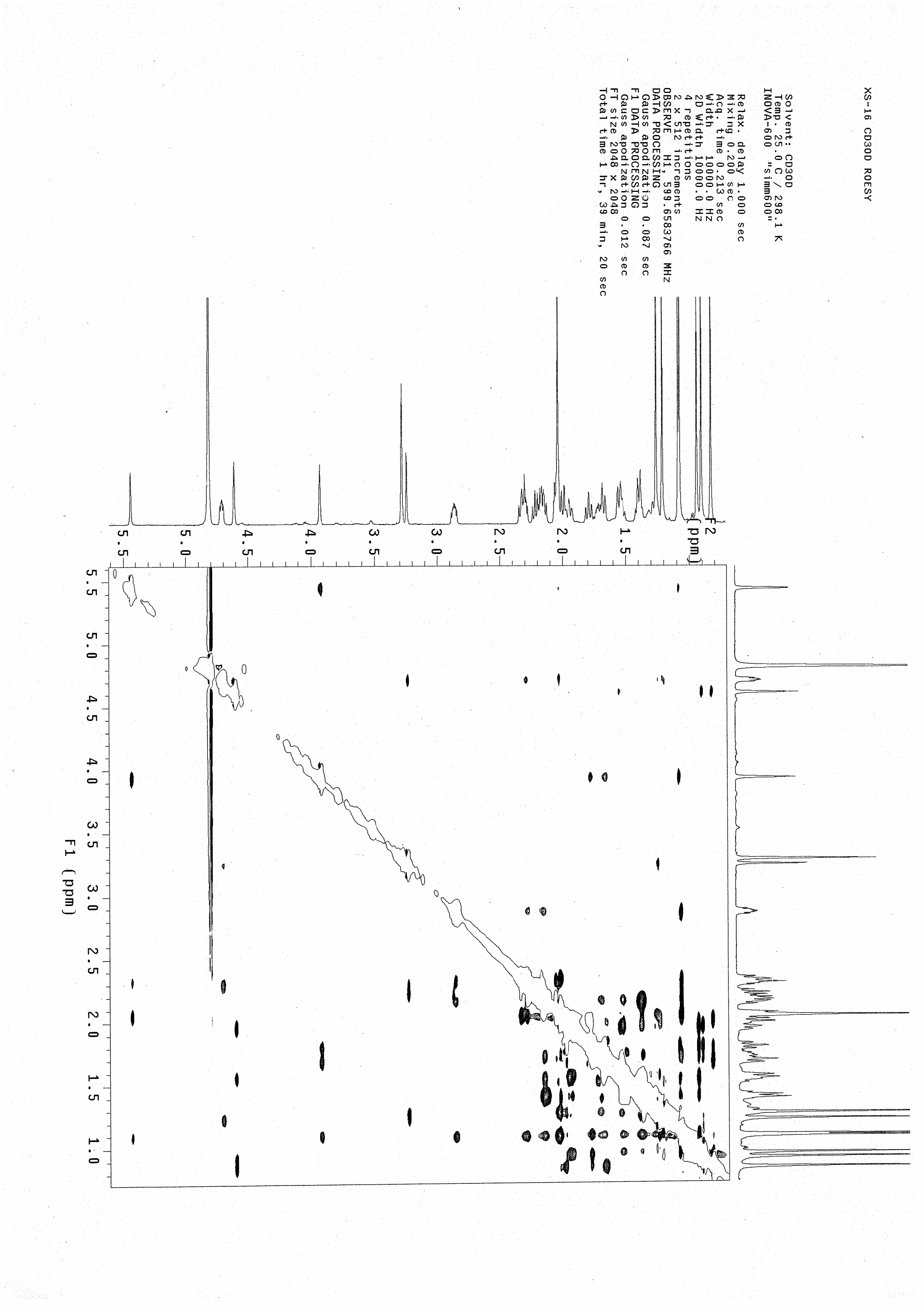

S7. ESIMS spectrum of chisiamol G (**1**)

Display Report

Analysis Info

Analysis Name	076-fi01.D	Acquisition Date	05/14/10 00:40:42
Method	Copy of SOPMSMSP.M	Operator	Administrator
Sample Name	yjm-XS-16	Instrument	esquire3000plus
Comment	?		

Acquisition Parameter

Ion Source Type	ESI	Ion Polarity	Positive	Alternating Ion Polarity	off
Mass Range Mode	Std/Normal	Scan Begin	100 m/z	Scan End	1750 m/z
Capillary Exit	158.5 Volt	Skim 1	40.0 Volt	Trap Drive	85.4
Accumulation Time	15000 鑑	Averages	3 Spectra	Auto MS/MS	on

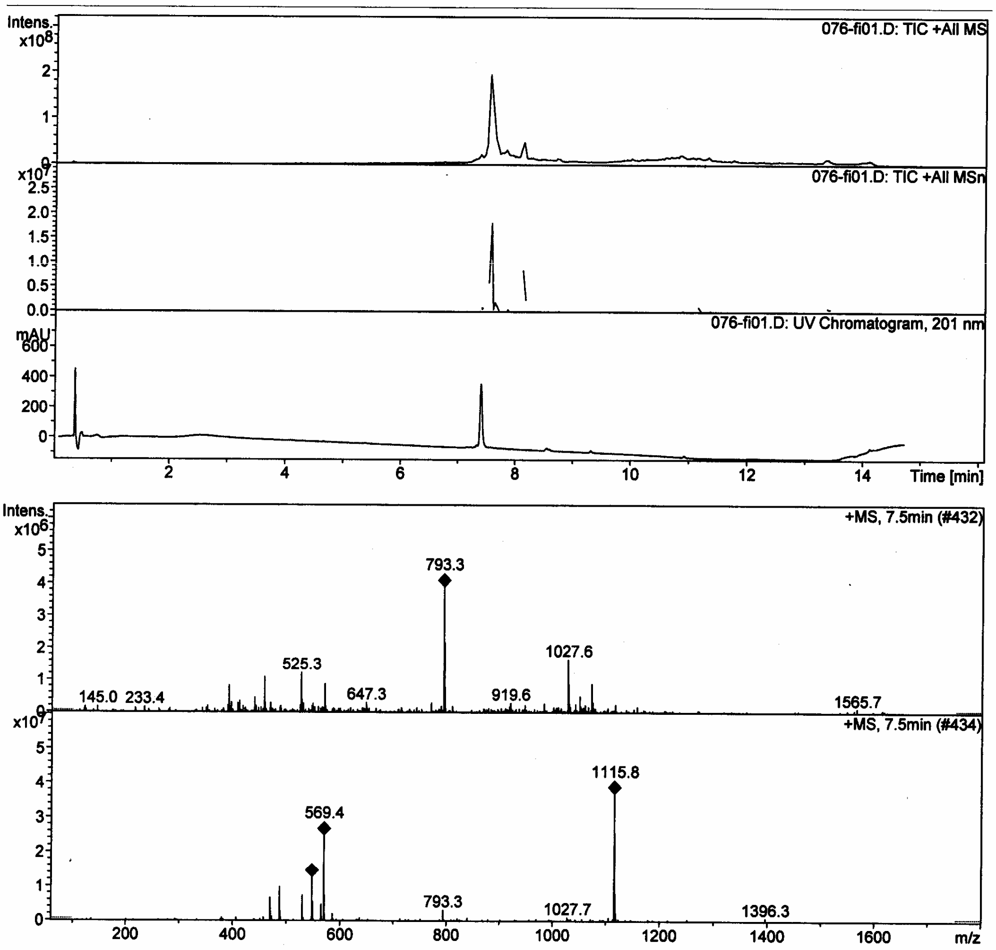

Bruker Daltonics DataAnalysis 3.1 printed: 05/14/10 09:45:24 Page 1 of 1

S8. HRESIMS spectrum of chisiamol G (**1**)

Mass Spectrum SmartFormula Report

Analysis Info

Analysis Name D:\Data\YUE\JYM-XS-16.d
Method tune_wide.m
Sample Name JYM-XS-16
Comment

Acquisition Date 6/1/2010 5:53:05 PM
Operator ECNU
Instrument / Ser# micrOTOF-Q 10198

Acquisition Parameter

Source Type	ESI	Ion Polarity	Positive	Set Nebulizer	0.4 Bar
Focus	Active	Set Capillary	4500 V	Set Dry Heater	180 °C
Scan Begin	50 m/z	Set End Plate Offset	-500 V	Set Dry Gas	4.0 l/min
Scan End	3000 m/z	Set Collision Cell RF	600.0 Vpp	Set Divert Valve	Source

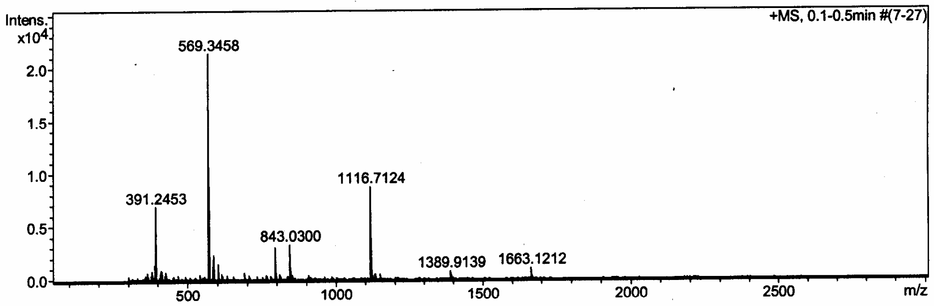

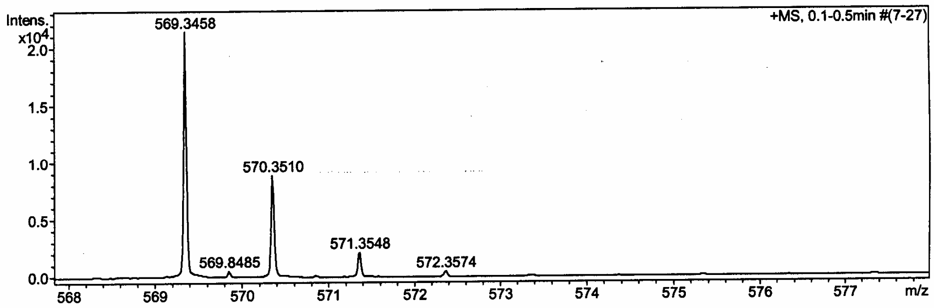

Meas. m/z	#	Formula	m/z	err [ppm]	Mean err [ppm]	N-Rule	e⁻ Conf	mSigma	Std I	Std Mean m/z	Std m/z Diff
569.3458											
	1	C 32 H 50 Na O 7	569.3449	-1.6	-2.7	ok	even	33.13	0.0511	0.0018	0.0018

Bruker Compass DataAnalysis 4.0 printed: 6/1/2010 6:01:12 PM Page 1 of 1

S9. IR spectrum of T chisiamol G (**1**)

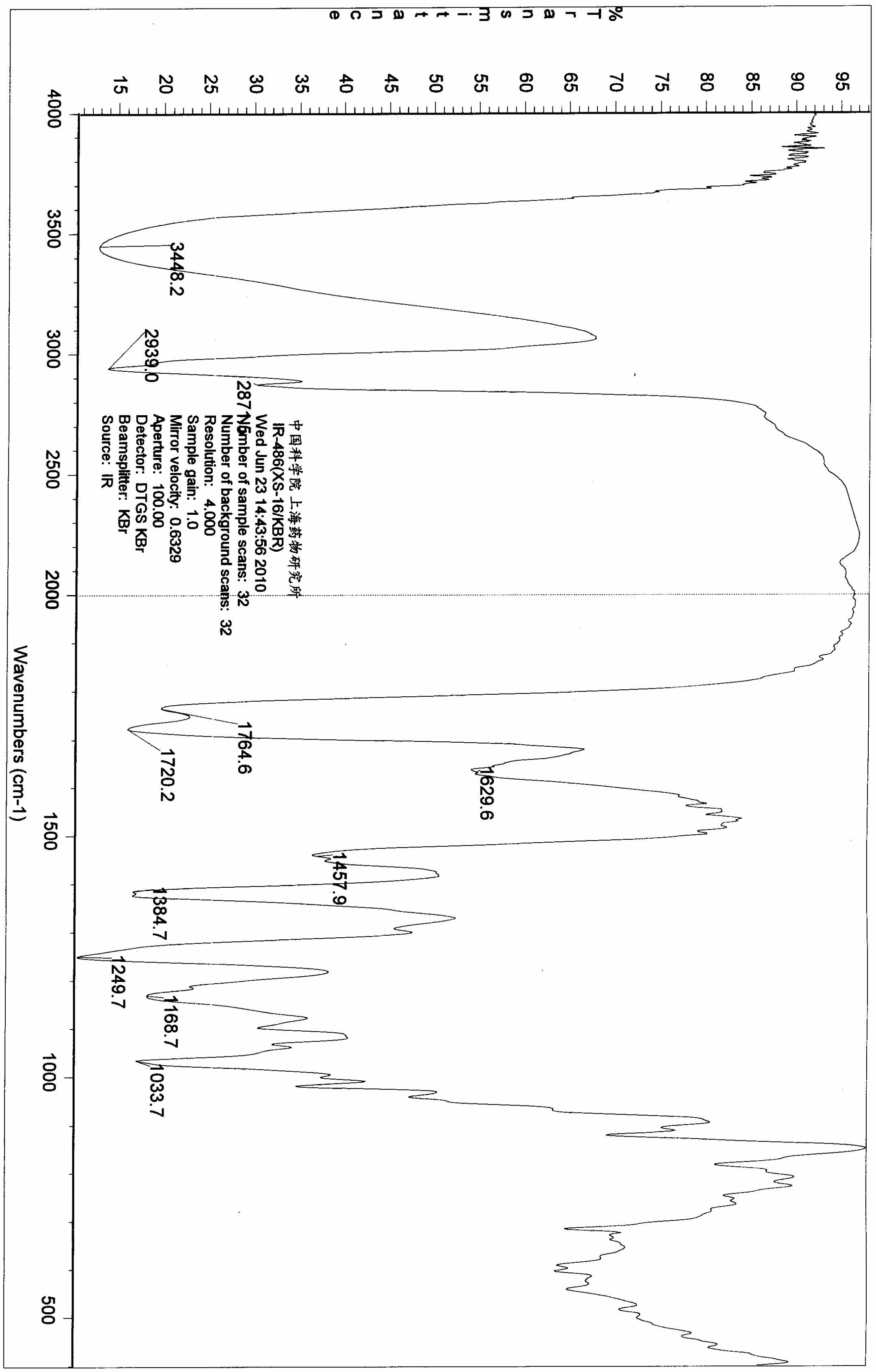

S10. ^{1}H spectrum of chisiamol H (**2**) in CD_3OD

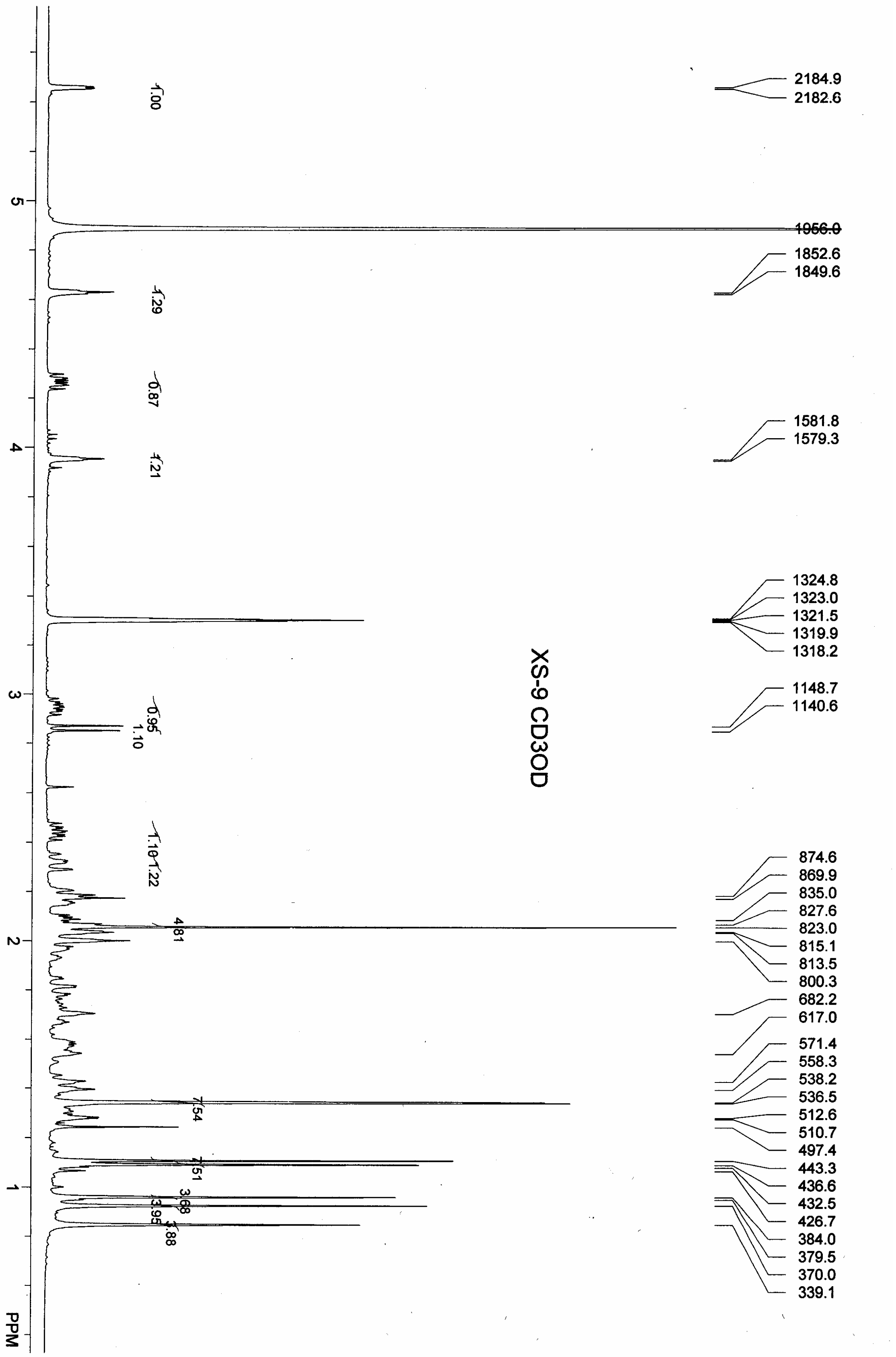

S11. ^{13}C spectrum of chisiamol H (**2**) in CD_3OD

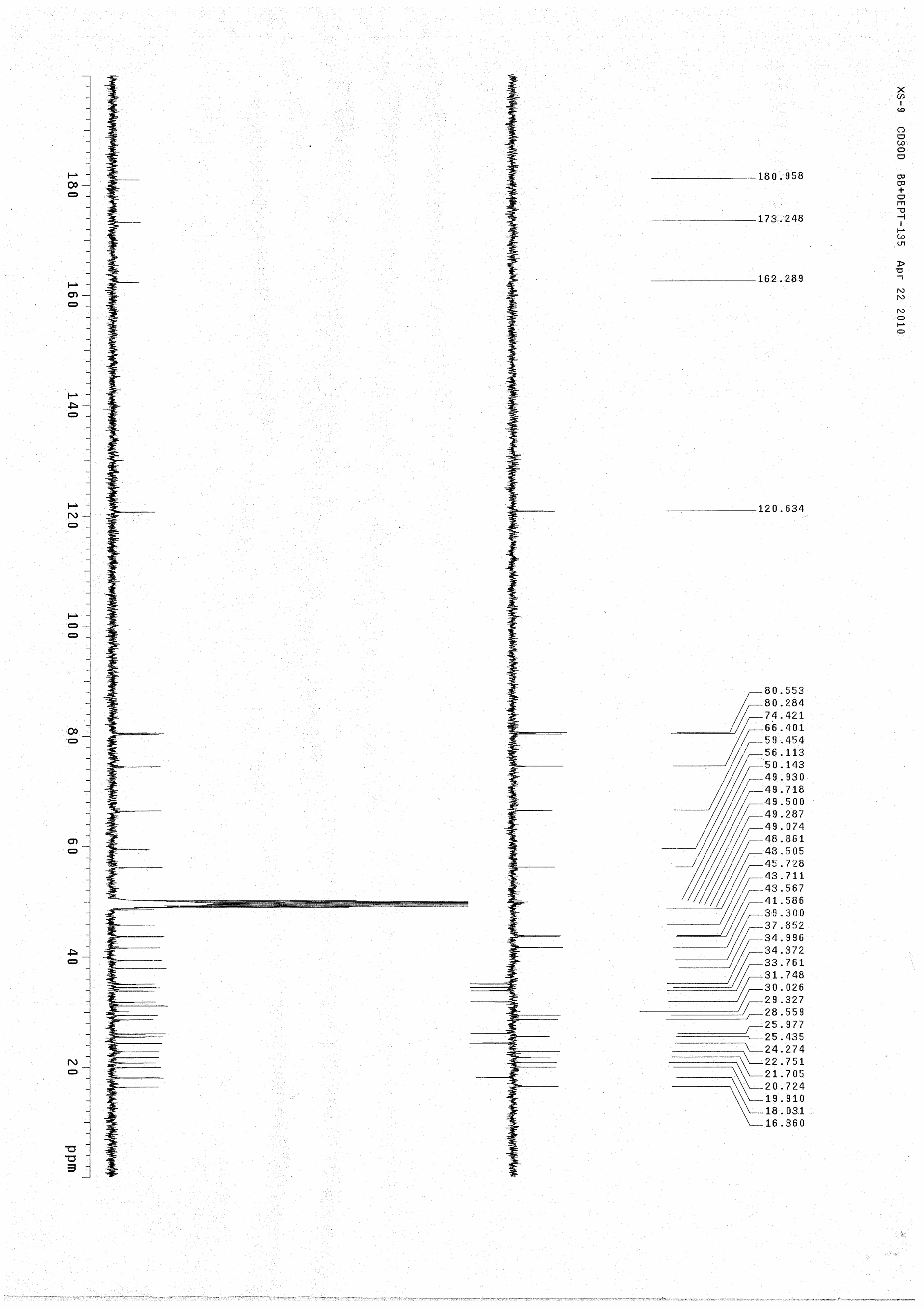

S12. HSQC spectrum of chisiamol H (**2**) in CD_3OD

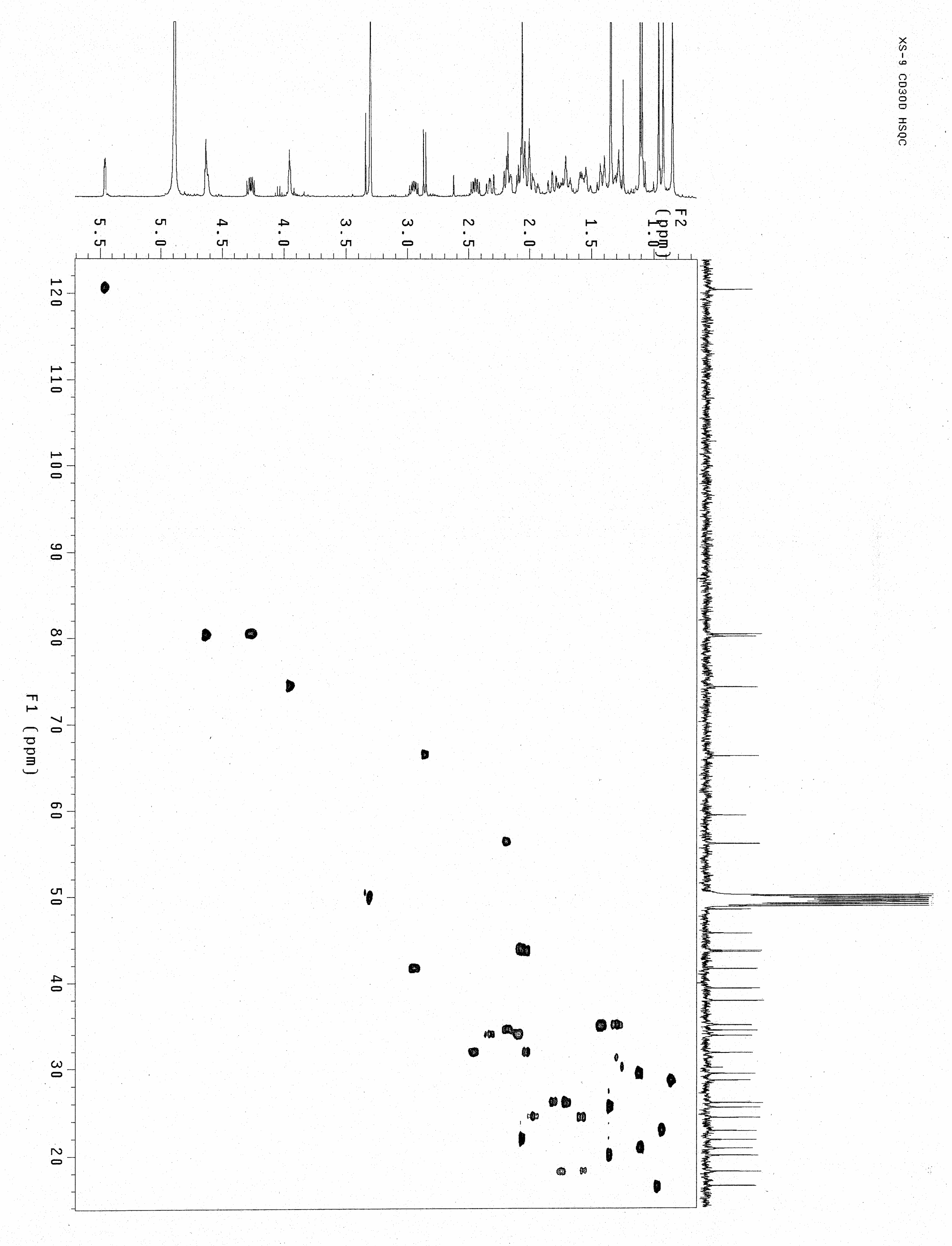

S13. HMBC spectrum of chisiamol H (**2**) in CD_3OD

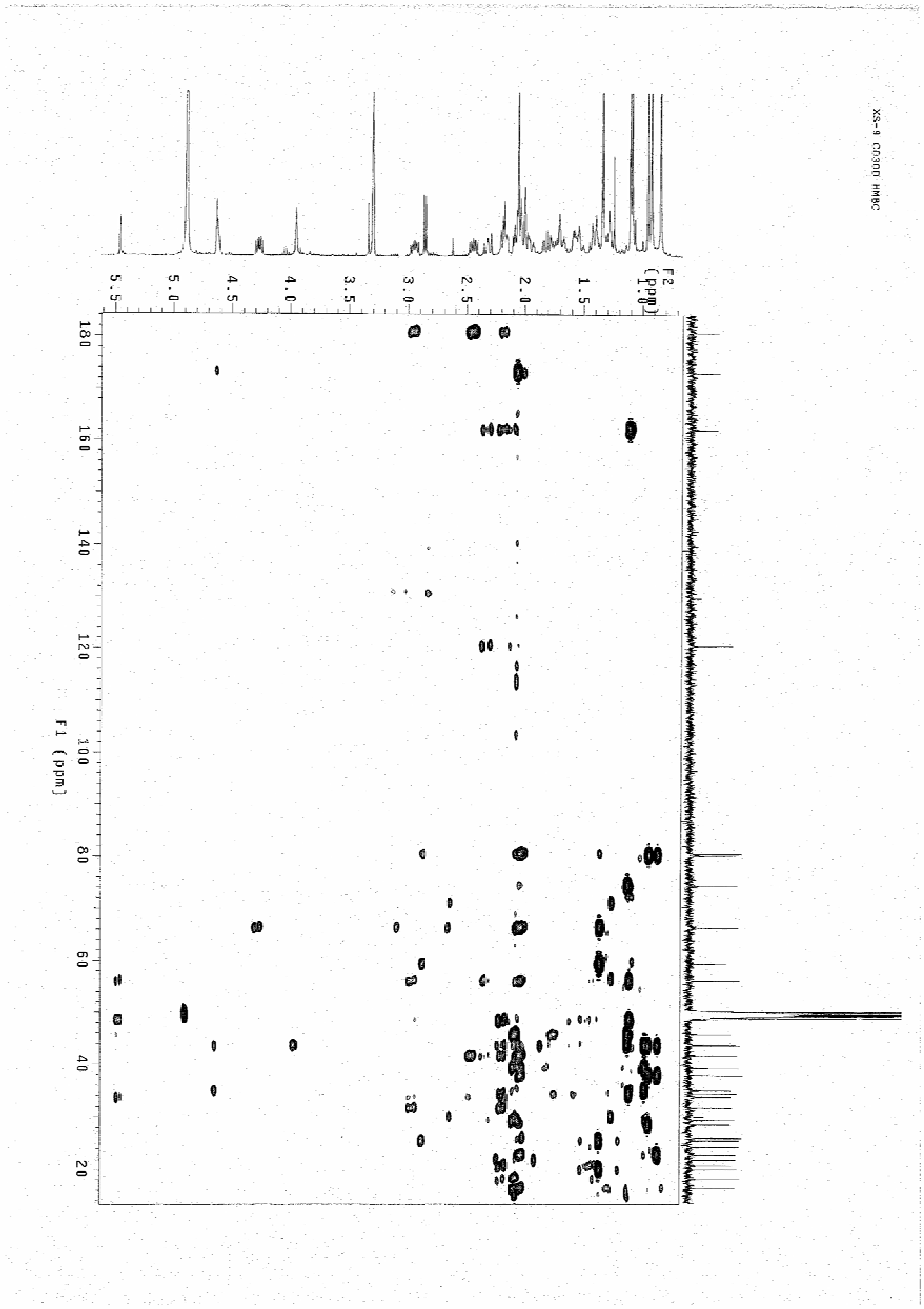

S14. COSY spectrum of chisiamol H (**2**) in CD_3OD

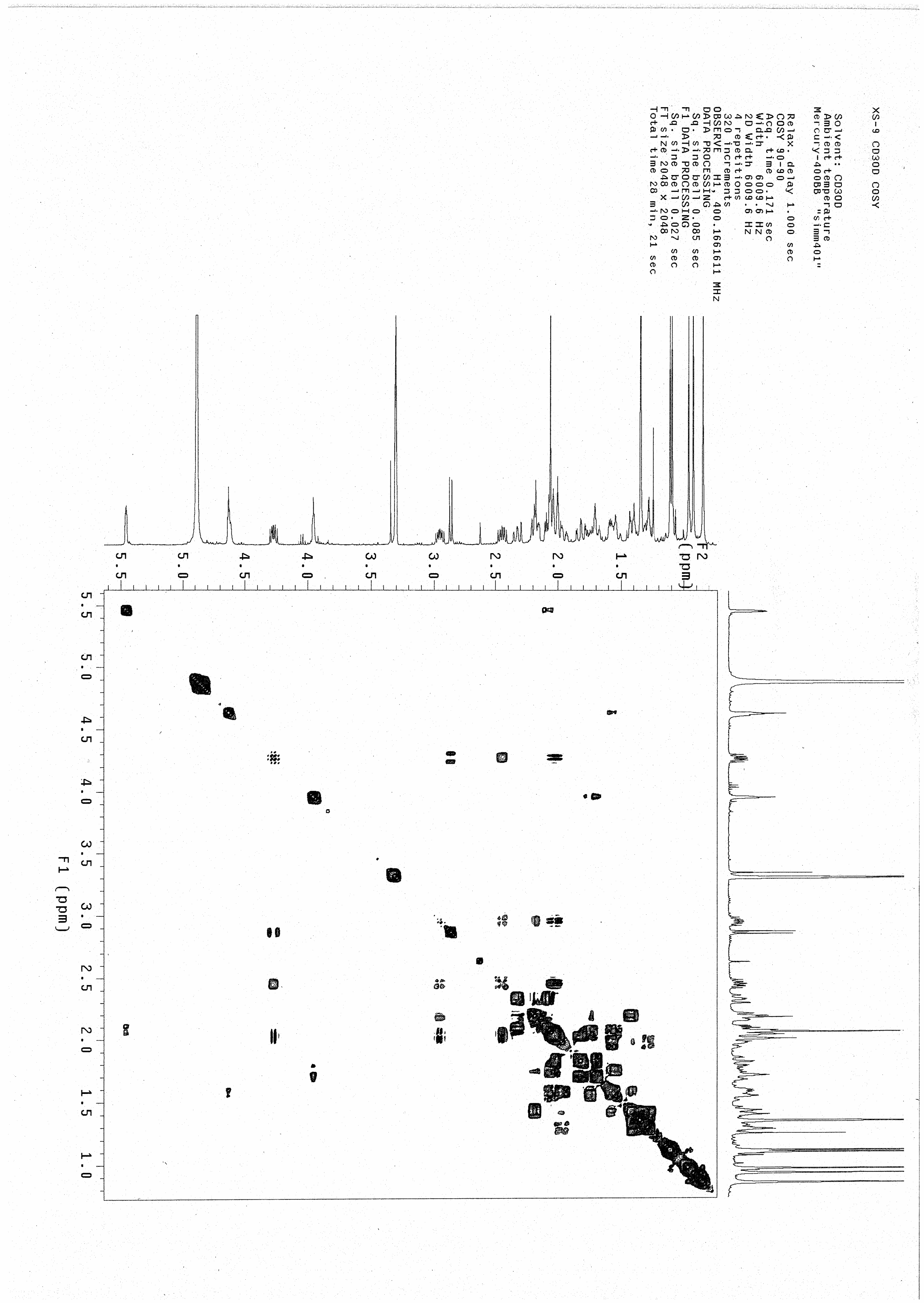

S15. ROESY spectrum of chisiamol H (**2**) in CD_3OD

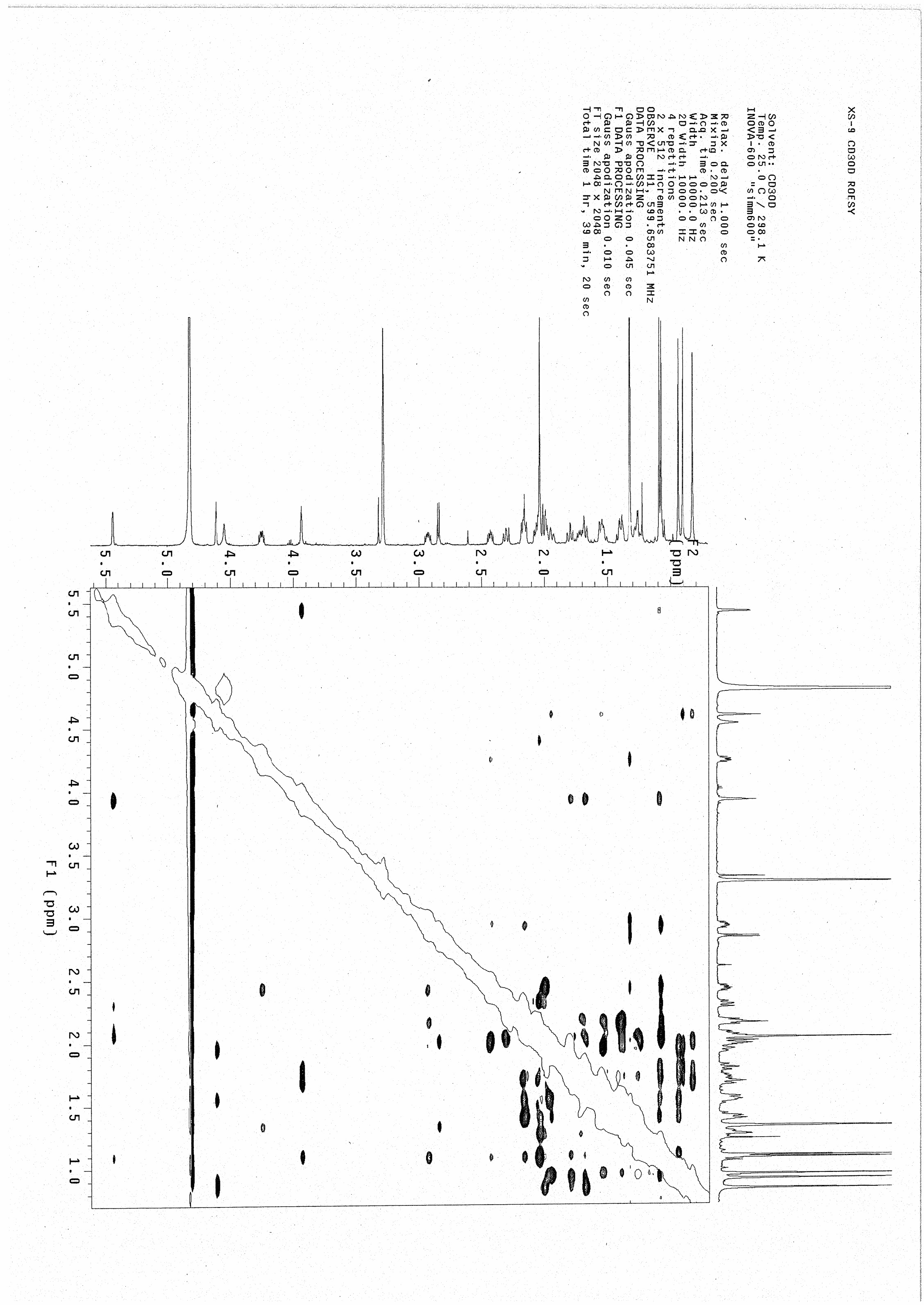

S16. ESIMS spectrum of chisiamol H (**2**)

Display Report

Analysis Info

Analysis Name	001-0401.D	Acquisition Date	05/18/10 15:01:50
Method	Copy of SOPMSMSP.M	Operator	Administrator
Sample Name	yjm-XS-9	Instrument	esquire3000plus
Comment			

Acquisition Parameter

Ion Source Type	ESI	Ion Polarity	Positive	Alternating Ion Polarity	off
Mass Range Mode	Std/Normal	Scan Begin	100 m/z	Scan End	1750 m/z
Capillary Exit	158.5 Volt	Skim 1	40.0 Volt	Trap Drive	85.4
Accumulation Time	8982 縦	Averages	3 Spectra	Auto MS/MS	on

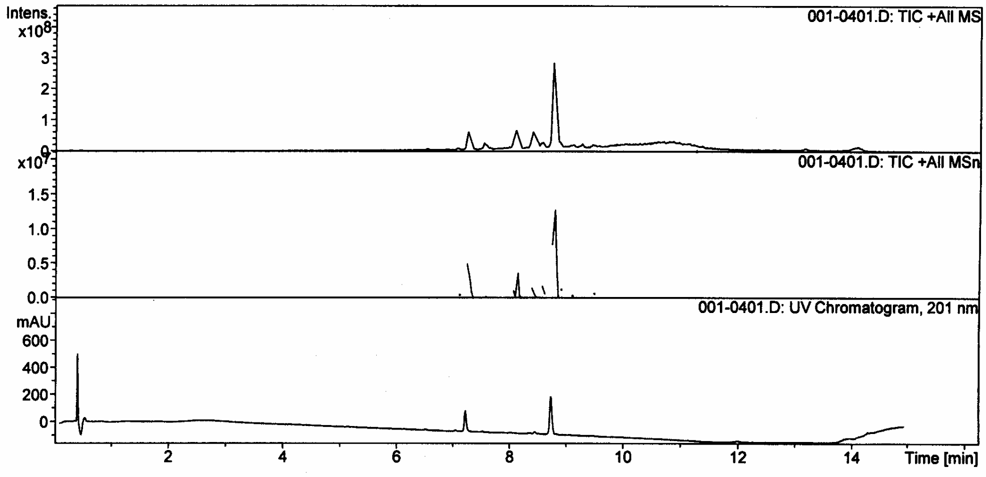

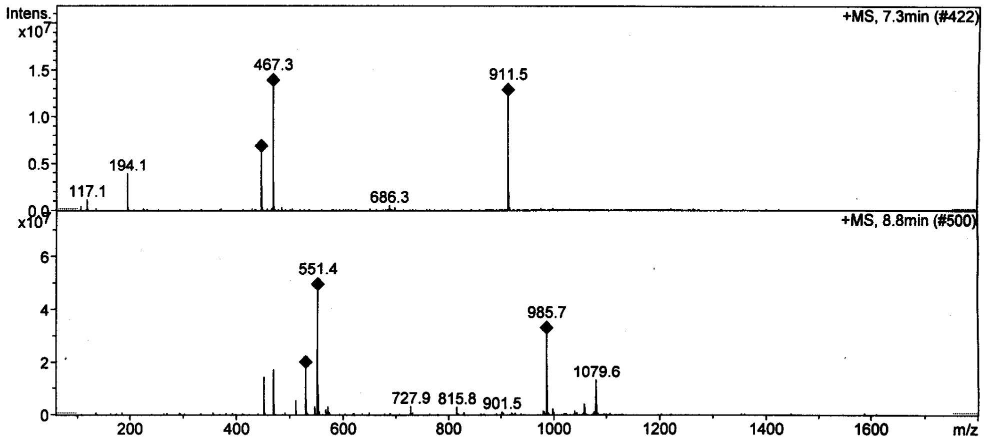

Bruker Daltonics DataAnalysis 3.1 printed: 05/19/10 12:59:31 Page 1 of 1

S17. HRESIMS spectrum of chisiamol H (**2**)

Elemental Composition Report **Page 1**

XS-9

Tolerance = 50.0 PPM / DBE: min = -1.5, max = 50.0
Isotope cluster parameters: Separation = 1.0 Abundance = 1.0%

Monoisotopic Mass, Odd and Even Electron Ions
12 formula(e) evaluated with 1 results within limits (up to 20 closest results for each mass)

100813-3 62 (1.170) AM (Cen,2, 80.00, Ht,9000.0,566.89,0.70); Sm (SG, 2x3.00); Cm (53:69) TOF MS ES+ 573

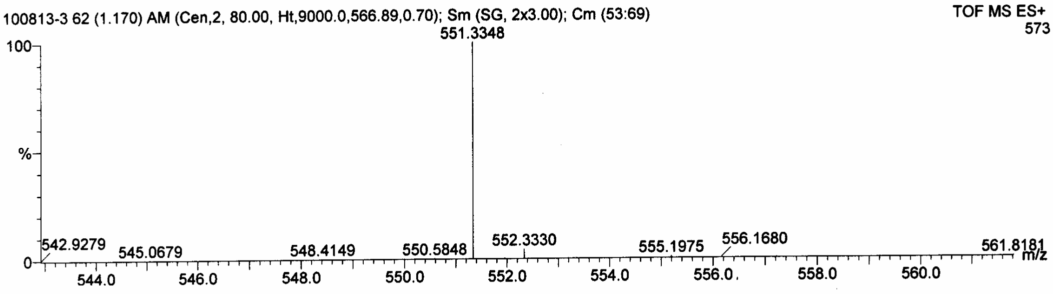

Minimum:	20.00				-1.5		
Maximum:	100.00		200.0	50.0	50.0		
Mass	RA	Calc. Mass	mDa	PPM	DBE	Score	Formula
551.3348	100.00	551.3349	-0.1	-0.1	8.5	1	C32 H48 O6 Na

S18. IR spectrum of chisiamol H (**2**)

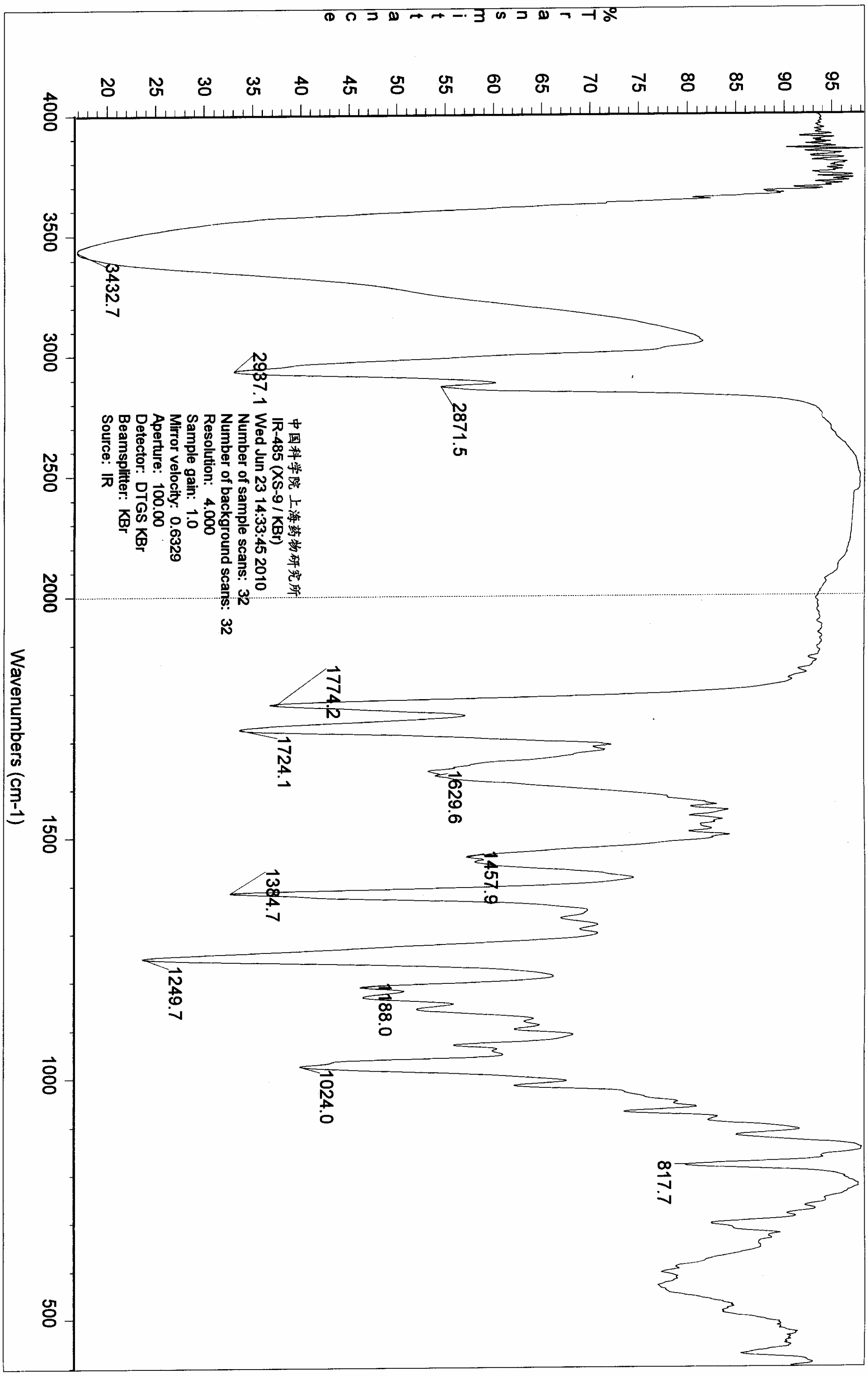

S19. ^{1}H spectrum of compound **8** in CD_3OD

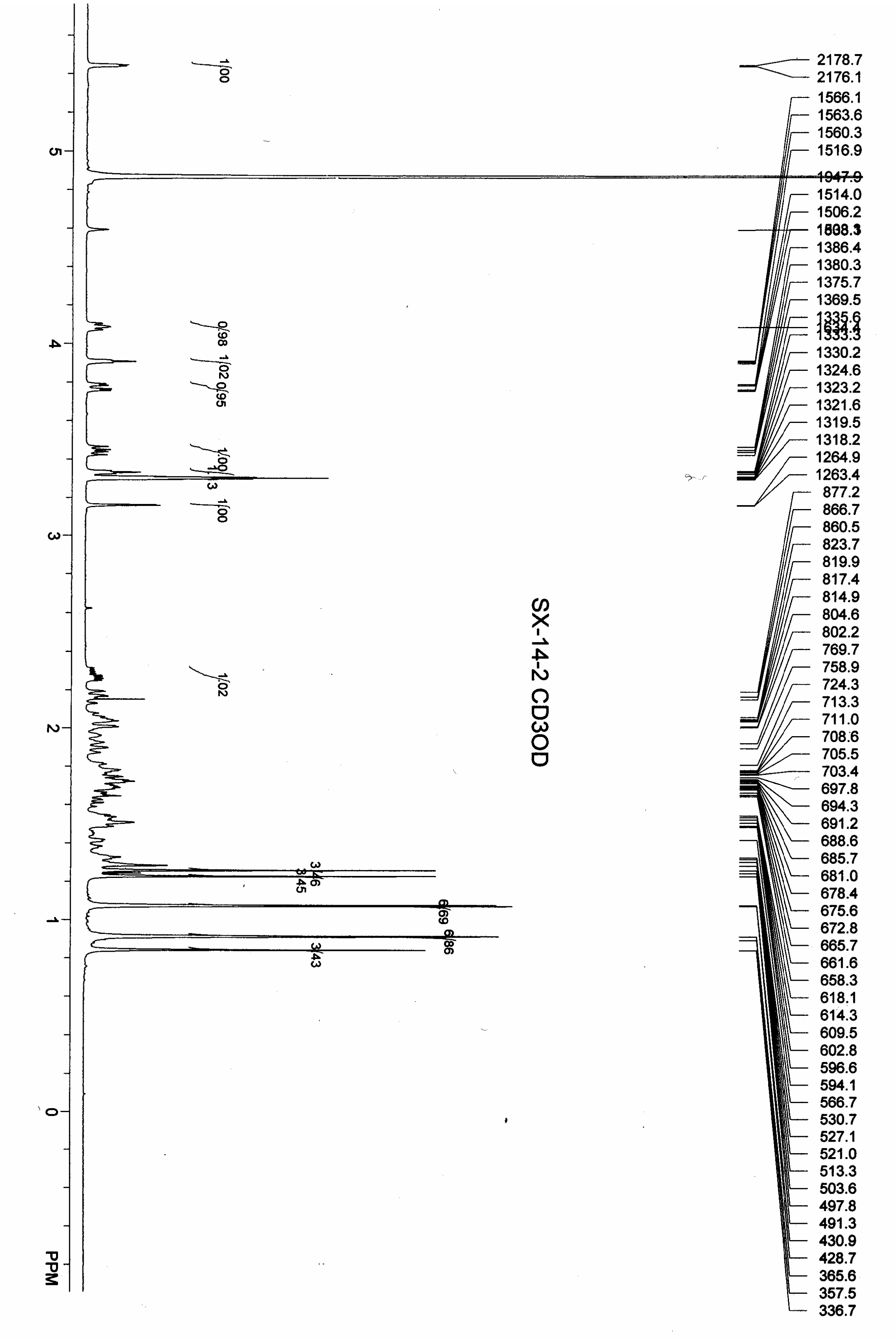

S20. ESIMS spectra of compound **8**

Display Report

Analysis Info

Analysis Name	046-ck01.D	Acquisition Date	08/26/10 14:42:08
Method	Copy of SOPMSMSP.M	Operator	Administrator
Sample Name	yjm-SX-14-2	Instrument	esquire3000plus
Comment	W		

Acquisition Parameter

Ion Source Type	ESI	Ion Polarity	Positive	Alternating Ion Polarity	off
Mass Range Mode	Std/Normal	Scan Begin	100 m/z	Scan End	1750 m/z
Capillary Exit	158.5 Volt	Skim 1	40.0 Volt	Trap Drive	85.4
Accumulation Time	9536 [illegible]	Averages	3 Spectra	Auto MS/MS	on

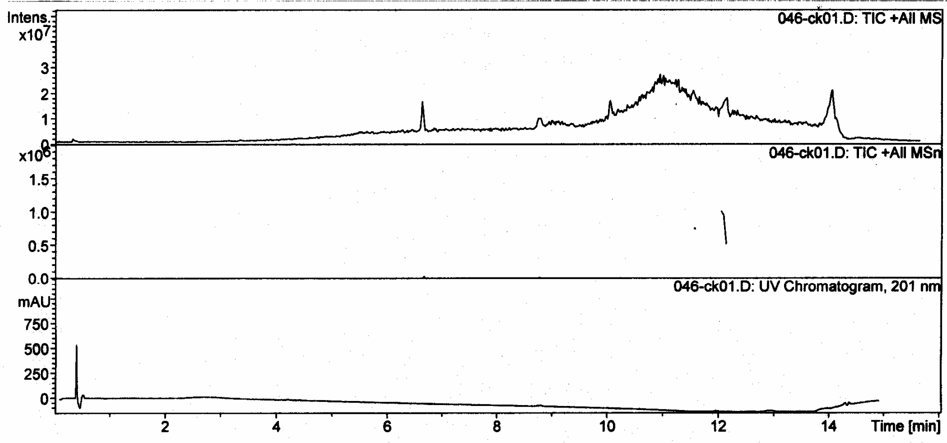

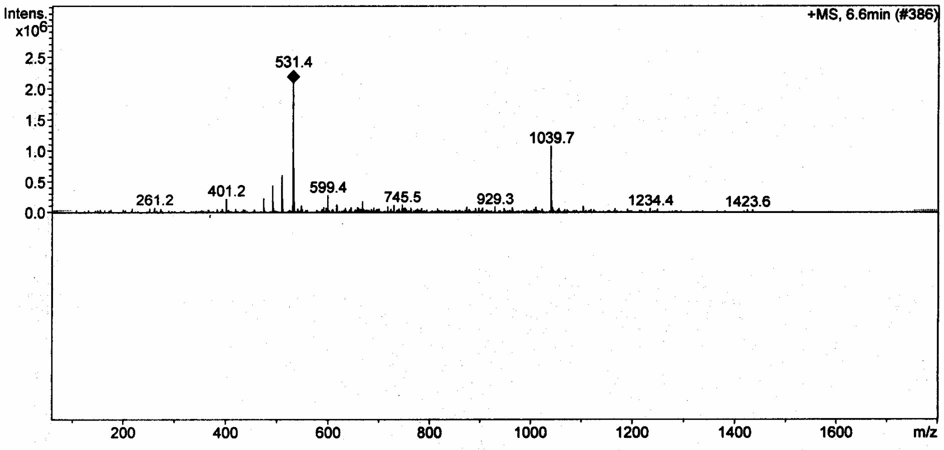

Bruker Daltonics DataAnalysis 3.1 printed: 08/27/10 09:18:58 Page 1 of 1

Display Report

Analysis Info

Analysis Name	046-cu01.D	Acquisition Date	08/26/10 17:28:48
Method	Copy of SOPMSMSN.M	Operator	Administrator
Sample Name	yjm-SX-14-2	Instrument	esquire3000plus
Comment	W		

Acquisition Parameter

Ion Source Type	ESI	Ion Polarity	Negative	Alternating Ion Polarity	off
Mass Range Mode	Std/Normal	Scan Begin	100 m/z	Scan End	1750 m/z
Capillary Exit	-158.5 Volt	Skim 1	-40.0 Volt	Trap Drive	92.9
Accumulation Time	9561 鏂	Averages	3 Spectra	Auto MS/MS	on

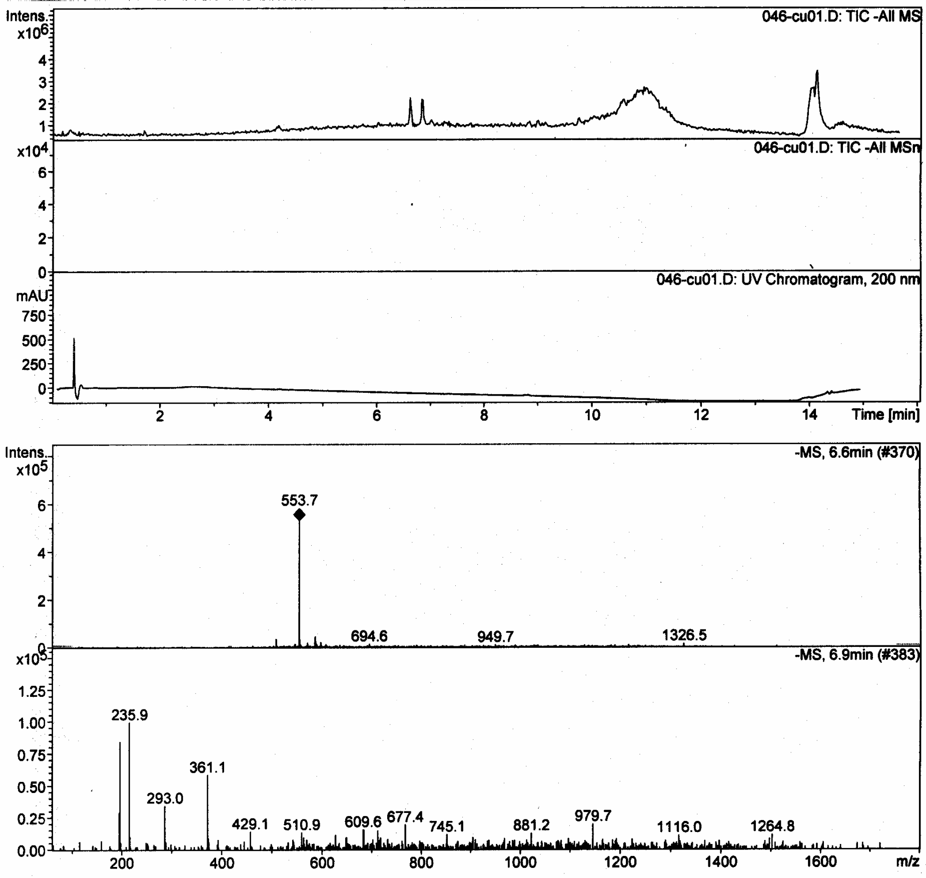

Bruker Daltonics DataAnalysis 3.1 printed: 08/27/10 09:19:02 Page 1 of 1
